# Effect of high-frequency (5Hz) rTMS stimulating left DLPFC combined with galantamine on cognitive impairment after ischemic stroke and serum homocysteine and neuron-specific enolase

**DOI:** 10.3389/fneur.2024.1345832

**Published:** 2024-02-28

**Authors:** Guojin Hu, Li Zhang, Xiuli Sun, Lin Wang, Qian Xu, Qin Li, Wei Huang, Yao Xiao

**Affiliations:** Department of Geriatric Rehabilitation, Shanghai Second Rehabilitation Hospital, Shanghai, China

**Keywords:** cognitive impairment after stroke, high-frequency repetitive transcranial magnetic stimulation, Galantamine, homocysteine, neuron-specific enolase

## Abstract

**Objective:**

To investigate the efficacy of high-frequency repetitive transcranial magnetic stimulation (HF-rTMS) combined with galantamine in patients with cognitive impairment after stroke and its effect on serum homocysteine (Hcy) and neuron-specific enolase (NSE) levels.

**Methods:**

A total of 90 patients with cognitive impairment after the first ischemic stroke were enrolled. They were randomly divided into rTMS+ cognitive rehabilitation group, Galantamine + cognitive rehabilitation group, and rTMS+ Galantamine + cognitive rehabilitation group. All groups received routine medical treatment and limb rehabilitation treatment. The rTMS stimulation site was the left dorsolateral prefrontal cortex (left DLPFC), the magnetic stimulation frequency was 5 Hz, the magnetic stimulation intensity was 80% of the motor threshold level, and 3,000 pulses were given every day. The Mini-Mental State Examination (MMSE), Montreal Cognitive Assessment (MoCA), Fugl-Meyer scale, and modified Barthel index, as well as rehabilitation scale and serum NSE and Hcy were evaluated before and after treatment (after 4 weeks).

**Results:**

After 4 weeks of treatment, the scores of MMSE, MoCa scale, Fugl-Meyer scale, and modified Barthel index in the three groups were significantly higher than those before treatment (all *p* < 0.05), while the serum NSE and Hcy levels of the three groups were decreased. rTMS+ Galantamine + cognitive rehabilitation group had higher scale scores, and the difference between the three groups was statistically significant compared with the other two groups (all *p* < 0.05).

**Conclusion:**

Cognitive rehabilitation combined with HF-rTMS and galantamine could improve the cognitive function of patients to the greatest extent, promote the recovery of physical activity, improve the self-care ability of daily life, and effectively reduce the serum HCY and NSE levels in patients with cognitive impairment after stroke. No randomized controlled trials of similar combination treatments have been reported. The better therapeutic effect may be related to the fact that galantamine combined with repetitive transcranial magnetism can activate the brain cholinergic system more extensively, promote brain neural remodeling through long-term potentiation and inhibit local neuroinflammatory responses in brain injury.

## 1 Introduction

Post-stroke cognitive impairment (PSCI), especially post-stroke dementia, seriously affects patients’ functional recovery, daily activities, and social functioning. PSCI is an independent risk factor affecting the prognosis of stroke. Professor Hachinski’s survey published in “Stroke” in 2006 showed that as many as 64% of stroke patients have varying degrees of cognitive impairment, and 1/3 will develop obvious dementia (1). A systematic review of epidemiological characteristics of post-stroke cognitive impairment in China in 2013 showed that the incidence rates of PSCI and post-stroke dementia (PSD) within 3 months after stroke were 56.6 and 23.2%, respectively (2). Compared with those without dementia, physical function of PSCI patients, their mental health status, and social functioning undergo more significant decline. Also, their functional independence is weakened, social participation ability is worsened, life satisfaction drastically decreases, and the 5-year survival rate is significantly lowered (3, 4). PSCI also seriously hinders the improvement of patients’ motor function, psychological state, self-care ability, and ability to participate in social activities and reduces life expectancy.

In recent years, non-invasive brain stimulation technology has developed rapidly, among which repetitive transcranial magnetic stimulation (rTMS) technology has received the greatest attention from researchers. rTMS acts on the central nervous system, mainly the brain, through a pulsed magnetic field, changing the membrane potential of neurons in the cerebral cortex, causing them to generate induced currents, affecting intramembrane metabolism and neural electrical activity, and inducing a series of physiological and biochemical changes. As a result, this technology has been widely investigated in recent years, producing positive effects on depression, cognitive impairment, post-stroke movement disorders, aphasia, etc. (5–7).

rTMS alters the excitability of cortical and subcortical neurons. Among them, high-frequency (>1 Hz) stimuli produce excitatory effects, and low-frequency (≤1 Hz) stimuli produce inhibitory effects. rTMS can act on synapses to produce long-term potentiation or long-term inhibitory effects and promote the excitation or inhibition of cortical neural circuits. Moreover, the physiological effects are persistent (8).

A large amount of available data indicates that TMS technology has a very unique role in the rehabilitation treatment of dementia, including degenerative dementias such as Alzheimer’s disease, as well as secondary dementias (mainly caused by vascular factors). Transcranial magnetic field can not only predict the risk of dementia in the brain, but also improve cognitive function by stimulating treatment to activate cholinergic neural pathways and promote brain injury remodeling. Existing transcranial magnetic studies have shown that patients with vascular dementia have increased motor cortex excitability (decreased resting motor threshold), which is consistent with patients with Alzheimer’s disease (9, 10). This may be part of a mechanism to compensate for plasticity after neuron loss and/or ischemic injury in the brain, with increased excitability helping to protect cognitive function. Abnormal resting motor threshold can be used as a “neurophysiological boundary point” to distinguish patients with normal cognition, non-demented vascular cognitive impairment, and vascular dementia (11). At the same time, the short-latency input inhibition measured by transcranial magnetic can reflect the function of cholinergic circuit in the central nervous system, which is closely related to cognitive function. Although there are some conflicting data in vascular dementia studies (12–14), this may be related to the variable location of subcortical infarctions in patients with vascular cognitive impairment and the significant differences in the distribution and extent of cholinergic denervation caused by them. Nevertheless, short latency inhibition holds great promise in the diagnosis and prognosis of different dementia processes and in the identification of acetylcholinesterase inhibitors for the treatment of sensitive individuals (15).

Transcranial magnetic therapy has a pleiotropic effect, although in a small number of experiments, the effects of high-frequency rTMS on mood, cognition, cortical microcircuits, neurotrophic/growth factors, and cerebral blood flow were not detected. This may be related to the selection of stimulus intensity, total pulse number, test sample size, sex selection, and the unclear mechanism of how ischemic brain injury affects plasticity induced by transcranial magnetic stimulation (16).

At present, there are many studies on the clinical application of rTMS in the treatment of post-stroke dementia, and gratifying results have been achieved (17, 18). Cha et al. (19) performed high-frequency transcranial magnetic therapy in patients with PSCI and assessed cognitive and emotional abilities at 2 and 14 weeks of treatment. The results showed that after rTMS treatment, the cognitive function of the patients was improved, and the proinflammatory cytokines in peripheral blood were decreased. These improvements lasted for three months. The meta-analysis by Li et al. (18) showed that the left dorsolateral prefrontal cortex of PSCI patients was stimulated by high-frequency rTMS, and the number symbol test, Rivermead behavioral memory test and the patients’ attention were significantly improved.

Galantamine hydrobromide is a second-generation cholinesterase inhibitor, initially used for the treatment of AD, which is highly selective for AChE in the central nervous system. It mainly inhibits cholinesterase at the synaptic cleft between the presynaptic membrane and the posterior membrane of brain cholinergic nerve cells, delays the degradation of acetylcholine, increases the content of available acetylcholine, stimulates and improves the function of the remaining acetylcholine receptors (20). The same time, after oral administration of Galantamine, the nicotinic cholinergic receptors in patients with vascular dementia can be regulated to a certain extent (allosteric regulation), and this change can promote a large amount of cholinergic nervous system in patients, releasing acetylcholine, promote its central nervous system into an excited state, and correct its memory and cognitive dysfunction. This unique dual action is beneficial for improving cognitive deficits in dementia patients.

Studies have shown that Galantamine has a good curative effect on patients with vascular dementia. In 2007, Auchus et al. (21) conducted a randomized, double-blind, and placebo-controlled clinical study involving multiple countries. Their results showed that the Galantamine group had significantly improved cognitive function and executive ability compared to the placebo group. In terms of improving activities of daily living, there was little difference between the two groups. The meta-analysis conducted by Yu-Dan et al. (22) showed that the treatment with donepezil and galantamine could significantly improve the Alzheimer’s disease cognitive scale (ADAS cog) score in patients with vascular dementia, with mild adverse reactions and high safety.

The treatment of PSCI requires early screening and detection and timely comprehensive intervention. Comprehensive intervention includes intervention and prevention of known risk factors, drug treatment, and rehabilitation. In addition, the recovery of cognitive function after stroke depends on repairing damaged nerve cells and cortical reconstruction, and intensive cognitive training can accelerate the process of cortical reconstruction. There is no ideal method for the treatment of PSCI, and the current treatment methods have poor efficacy. We attempted to combine high-frequency repetitive transcranial magnetic stimulation and galantamine oral therapy on the basis of cognitive rehabilitation therapy for PSCI patients, achieving better efficacy than the control group. The following report is presented.

## 2 Methods

### 2.1 Subjects

**Figure 1:**
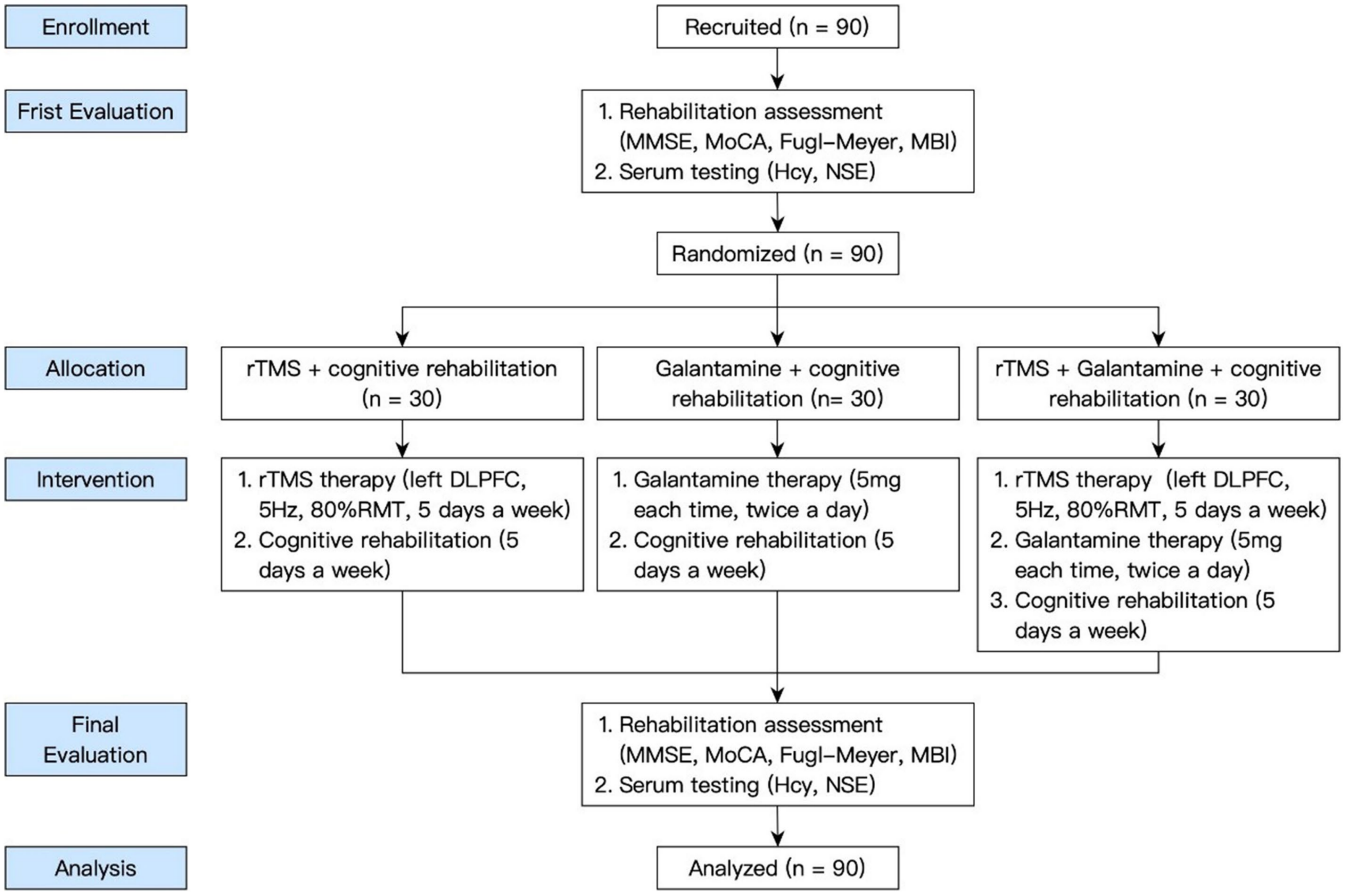
The flow-chart of the study.

By posting recruitment advertisements in the hospital lobby, trial participants were recruited from inpatients in Shanghai Second Rehabilitation Hospital from January 2021 to June 2022. When a patient voluntarily participated in the trial, it was judged whether he or she met the inclusion and exclusion criteria. Patients who met the criteria was randomly assigned to each group after rehabilitation evaluation. The general information of all the patients was collected, the case report form was filled out, and the data files were established. The research flow chart is shown in Figure 1.

Inclusion criteria were the following: ① clinical manifestations met the diagnostic criteria for ischemic stroke revised by the 4th National Cerebrovascular Disease Academic Conference (23), all of which were the first onset, and the onset time was within 6 months; ② diagnosed with vascular dementia according to criteria for vascular dementia listed in the “2016 Guidelines for the Diagnosis and Treatment of Vascular Cognitive Impairment in China” (24), and the cognitive impairment affected the patient’s ability to perform activities of daily living. The main points of diagnosis included the following 3 aspects: a. cognitive dysfunction: patients complaining of cognitive dysfunction, or informants reporting that the patient had cognitive impairment, and objective examination suggested that the patient had evidence of cognitive impairment, and (or) objective examination confirmed that the patient’s cognitive function was relatively weak; b. vascular factors: risk factors for cerebrovascular disease, history of stroke, focal signs of the nervous system, and evidence of cerebrovascular disease provided by neuroimaging; c. there was a causal relationship between cognitive dysfunction and vascular factors. ③ Aged 50 to 80 years old; ④ with a correct understanding of the significance of this study and good compliance; the subjects who voluntarily participated in the clinical trial, signed an informed consent form, and completed relevant treatment and examination.

Exclusion criteria were the following: ① previous cognitive impairment or cognitive decline caused by other neurological diseases, such as Alzheimer’s disease (AD), Parkinson’s disease, and Parkinson’s syndrome; ② concurrent with other serious physical diseases, mental implants such as cardiac pacemakers installed in the body, or skull defects; ③ patients with severe visual, hearing and language impairments; ④ cerebral hemorrhage, history of mental illness, history of epilepsy, etc.; ⑤ those with a serious medical disease; ⑥ patients with malignant tumors.

Using the minimum sample size estimation method (a two-sided test with a confidence level of 95% and an allowable error of 5%), the experimental sample size was determined. Patients with the first ischemic stroke were divided into 3 groups (30 cases in each group): rTMS+cognitive rehabilitation group (rTMS group), Galanta Min + cognitive rehabilitation group (Galantamine group), and rTMS+Galantamine+cognitive rehabilitation group (combined group). The general information about the patients was collected, the case report form was filled out, and the data files were established.

Since PSCI has clear and effective treatment methods such as cholinesterase inhibitors or rTMS, it is a long-term chronic process with a small placebo effect, so we did not set up a pseudo-stimulation or placebo group in our study. This study was approved by the Ethics Committee of Shanghai Second Rehabilitation Hospital. Written informed consent was obtained from all patients.

### 2.2 Evaluation indicators

#### 2.2.1 Rehabilitation assessment

Patients were evaluated using MMSE, MoCA scale, Fugl-Meyer motor scale, and modified Barthel index.

##### 2.2.1.1 The Mini-Mental State Examination

MMSE compiled by Folstein et al. (25), is a simple and easy-to-use tool most commonly used to assess cognitive function. It can screen patients with dementia and judge the severity of cognitive impairment. The scale includes orientation, memory, attention and calculation, recall ability, language ability, etc., with a total score of 30 points. The lower the score on this scale, the poorer the cognitive function.

##### 2.2.1.2 Montreal Cognitive Assessment

MoCA was compiled by Nasreddine et al. (26) at the Neurology Clinical Research Center of Charles LeMoye Hospital in Canada. It includes tests in 8 cognitive domains: memory function, visuospatial function, executive function, attention, calculation, language function, time orientation, and place orientation. The highest score is 30 points, and ≥ 26 is considered normal.

##### 2.2.1.3 Simplified Fugl-Meyer motor function evaluation scale

We used a quantitative evaluation method designed by Swedish scholar Fugl Meyer mainly based on Brunnstrom’s point of view (27). This approach is widely used in the clinical assessment of movement disorders in stroke patients. The highest motor function score is 66 for the upper limbs and 34 for the lower limbs. After extensive testing, the scale assesses scores with good consistency, responsiveness, and accuracy.

##### 2.2.1.4 Modified Barthel index

This scale was jointly compiled by Mahoney and Barthel in 1965 and is used to evaluate patients’ activities of daily living, including eating, bathing, grooming, dressing, urinating/urinating control, using the toilet, and bed and chair transfer and 10 items of walking on flat ground and going up and down stairs. It is suitable for people with impaired daily life abilities caused by various reasons (28). The total score is 100 points. The higher the score, the stronger the ability to take care of oneself.

#### 2.2.2 Serum testing

##### 2.2.2.1 Homocysteine

Hcy is an intermediate product of methionine metabolism. Under physiological conditions, its plasma concentration is maintained at a low level, while it increases during certain conditions, e.g., diabetes, coronary heart disease, cerebrovascular disease, etc. Hcy is considered a risk of morbidity and is an independent risk factor for dementia (29). In this study, the whole blood was drawn from the patients in the morning and sent to the laboratory to detect the level of Hcy by circulating enzyme method.

##### 2.2.2.2 Neuron-specific enolase

NSE is specifically distributed in nerve tissue and neuroendocrine cells. When nerve cells are injured by ischemia, the permeability of the nerve cell membrane changes and NSE can be released from the cells into the peripheral blood circulation. Therefore, the level of serum NSE is a sensitive biochemical index for judging the degree of brain nerve damage in patients with VaD (30). In addition, the degree and prognosis can have an effective predictive role. In this study, the NSE level in serum was analyzed by enzyme-linked immunosorbent assay (ELISA).

### 2.3 Medical drug therapy

The patients in the three groups received routine medical treatment (such as lowering blood pressure, controlling blood sugar, regulating lipid metabolism, nourishing cranial nerves and anticoagulation, etc.) and were not given drugs that could affect cognitive function. Central nervous system active drugs such as rivastigmine, tacrine and so on affect the brain physiological activity index monitored by transcranial magnetic stimulation (31–35). In our trial, with the exception of galantamine, patients did not use these drugs, which have been reported and may affect the TMS markers.

### 2.4 Routine rehabilitation treatment

The three groups of patients were all given routine rehabilitation training, and the rehabilitation training content included: (1) motor function training of the limbs on the hemiplegic side, such as body position placement, passive joint movement, bedside, and bedside training, sitting and standing training, walking training inside parallel bars, walking training, upper limb functional training, etc.; (2) daily living training, including dressing training, eating training, living training, behavior change, and personal hygiene training, etc.

### 2.5 Cognitive training

Cognitive training mainly included following 7 parts: ① attention training: stimulus–response method was applied (which includes comparing the similarities and differences between two images, finding the same graphics as the target card among similar graphics, retelling the story after listening to the story, deleting specified letters, etc.); ② orientation training: calendars, reminder cards, etc., were used to train patients’ time orientation, and gather pictures or photos of public figures, patients’ family members, and friends for patients to identify repeatedly; ③visual–spatial structural ability training: canceling homework, prism therapy, copying clocks or matchstick shapes, copying various plane and three-dimensional figures, etc.; ④ memory training: oral memory methods (such as making up stories, associating methods, and PQRST method), visual image technology (such as “memory map” method, etc.), and using memory aids (such as diaries, memos, etc.) to assist patients complete memory tasks in everyday life; ⑤ computational training: including digital cognition, digital games or homework, etc.; ⑥ executive function and problem-solving ability training: finding out the required information from newspapers, arranging numbers training, item classification training, budgeting training and hypothetical problem handling, etc., and asking patients to try to apply this training to their daily life; ⑦ language and communication skills training: communicating more with patients, making gradual progress, and carrying out targeted training according to the type of language communication barriers of patients.

The above-mentioned cognitive training was carried out by the therapist according to the patient’s cognitive impairment, using one or several of aspects as appropriate, once a day, 60 min each time, 5 days a week, for 4 weeks.

### 2.6 Galantamine usage

The Galantamine+cognitive rehabilitation group and the rTMS+Galantamine+cognitive rehabilitation group received galantamine hydrobromide dispersible tablets (Zhejiang Yixin Pharmaceutical Co., Ltd) twice a day, 5 mg each time, for a total of 4 weeks.

### 2.7 Repeated transcranial magnetic therapy

The rTMS+cognitive rehabilitation group and the TMS + Galantamine+cognitive rehabilitation group were treated with rTMS supplemented by the CCY-I type transcranial magnetic stimulator produced by Wuhan Yirad.

The patient’s cortex’s resting motor threshold (RMT) was measured during the first treatment. Briefly, the patient was placed in a sitting or supine position, and the cortex of the right-handed thumb motor area (M1) was stimulated in a single-pulse mode for 10 times, 5 of which were used to induce the movement of thumb abductor muscle, and the energy of the stimulation intensity was RMT. An international standard EEG electrode 10–20 lead system was used to locate the active part of the coil. A circular magnetic stimulation coil was selected; the center point of the coil was tangent to the patient’s scalp surface, the stimulation site was the dorsolateral area of the left frontal lobe (left DLPFC), the magnetic stimulation frequency was 5 Hz, the magnetic stimulation intensity was 80% of the motor threshold level, and 3,000 pulses were given every day (36).

Transcranial magneto therapy was started every day 10 min after the end of the cognitive training, 5 days a week, 5 days of treatment as a course of treatment, with an interval of 2 days between each course of treatment, a total of 4 courses of treatment. During the treatment, the therapist monitored whether there were any adverse reactions during the treatment.

### 2.8 Final evaluation

Before and after the treatment, an independent evaluator conducted a rehabilitation evaluation using MMSE, MoCA scale, Fugl-Meyer scale, and modified Barthel index. The evaluator was blinded to the grouping. Also, adverse reactions during the course of treatment were recorded, and serum Hcy and NSE levels were analyzed.

### 2.9 Statistical analysis

SPSS19.0 was used for statistical processing, the comparison of count data was carried out by χ^2^ test, the measurement data were expressed by χ ± s, and the normality and homogeneity of variance tests were carried out, and the t-test of two independent samples was used for comparison between two groups. One-way analysis of variance was used for comparison between groups, and a non-parametric rank sum test was used for data that did not conform to a normal distribution and homogeneous variance. Pearson correlation analysis was used to analyze the correlation between cognitive function, motor function, and activities of daily living. *p* < 0.05 indicated statistically significant difference.

## 3 Results

### 3.1 Comparison of the general data of the three groups of patients

There was no significant difference in age, gender, average onset months, lesion side, and stroke type among the three groups (all *p* > 0.05) (Table 1).

**Table 1:** Comparison of general information among three groups.

Group	Number	Gender	Age (year)	Hemiplegic side	A course of disease (month)
		Male/female		Left/right	
rTMS group	30	12/18	68.00 ± 9.01	18/12	3.46 ± 1.56
Galantamine group	30	15/15	63.43 ± 8.42	11/19	3.25 ± 1.79
combined group	30	11/19	65.03 ± 8.60	16/14	3.20 ± 1.60
*χ^2^/F* value		1.18	2.14	3.47	0.22
*p* value		0.55	0.12	0.18	0.81

### 3.2 Comparison of MMSE and MoCA scores in the three groups before and after treatment

**Figure 2:**
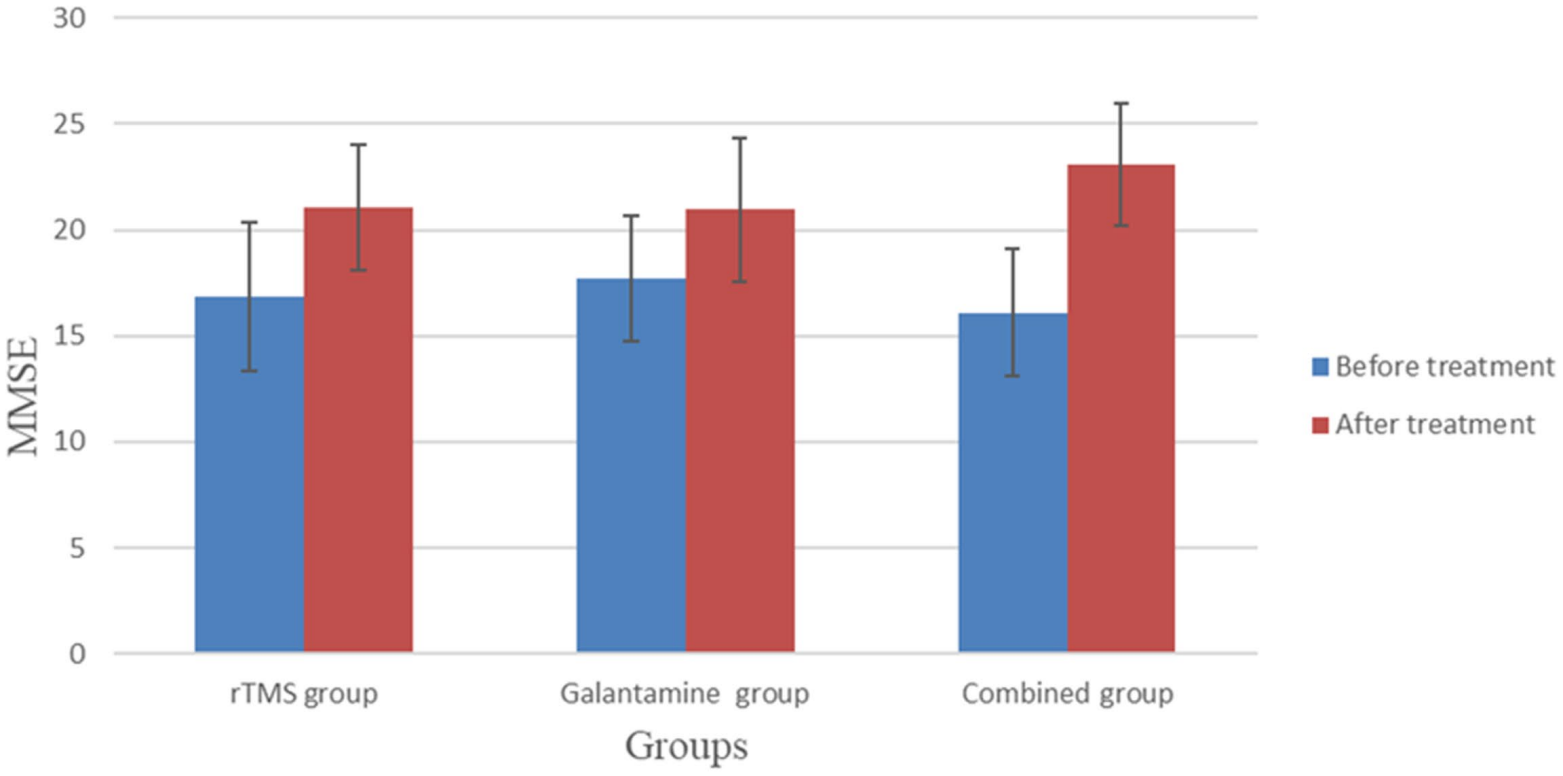
Comparison of MMSE between the two groups before and after treatment.

**Figure 3:**
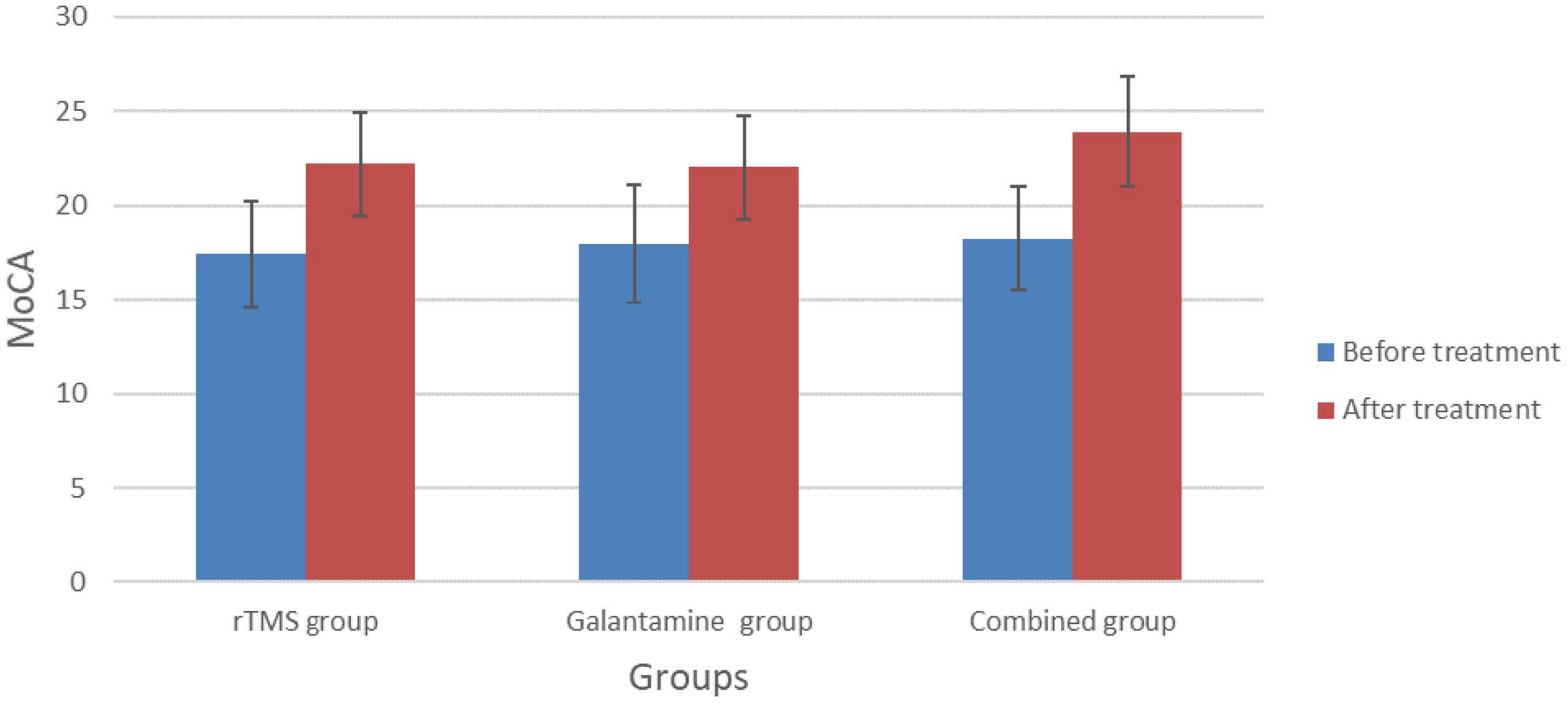
Comparison of MoCA scores between the three groups before and after treatment.

Before treatment, there was no significant difference in MMSE and MoCA scores among the three groups (*p* > 0.05). However, after 4 weeks of treatment, the MMSE and MoCA scores of the three groups were all higher than those before treatment (all *p* < 0.05). After 4 weeks of treatment, there was a statistically significant difference in MMSE and MoCA scores among the three groups (*p* < 0.05), with the combined group having the highest score (Table 2; Figures 2, 3).

**Table 2:** Comparison of MMSE and MoCA scores between the three groups before and after treatment (points, $\bar{x}\pm s$ ).

Group	Number of cases	MMSE		MoCA	
		Before treatment	After treatment	Before treatment	After treatment
rTMS group	30	16.83 ± 3.53	21.07 ± 2.95^a^	17.43 ± 2.82	22.23 ± 2.74^a^
Galantamine group	30	17.70 ± 2.94	20.97 ± 3.39^a^	18.00 ± 3.12	22.03 ± 2.77^a^
Combined group	30	16.10 ± 3.03	23.07 ± 2.91^a^	18.27 ± 2.72	23.93 ± 2.94^a^
*χ^2^/F* value		1.90	4.41	0.65	4.12
*p* value		0.16	0.02	0.53	0.02

a Compared with before treatment, *p* < 0.05.

### 3.3 Comparison of Fugl-Meyer scale scores before and after treatment in the three groups

**Figure 4:**
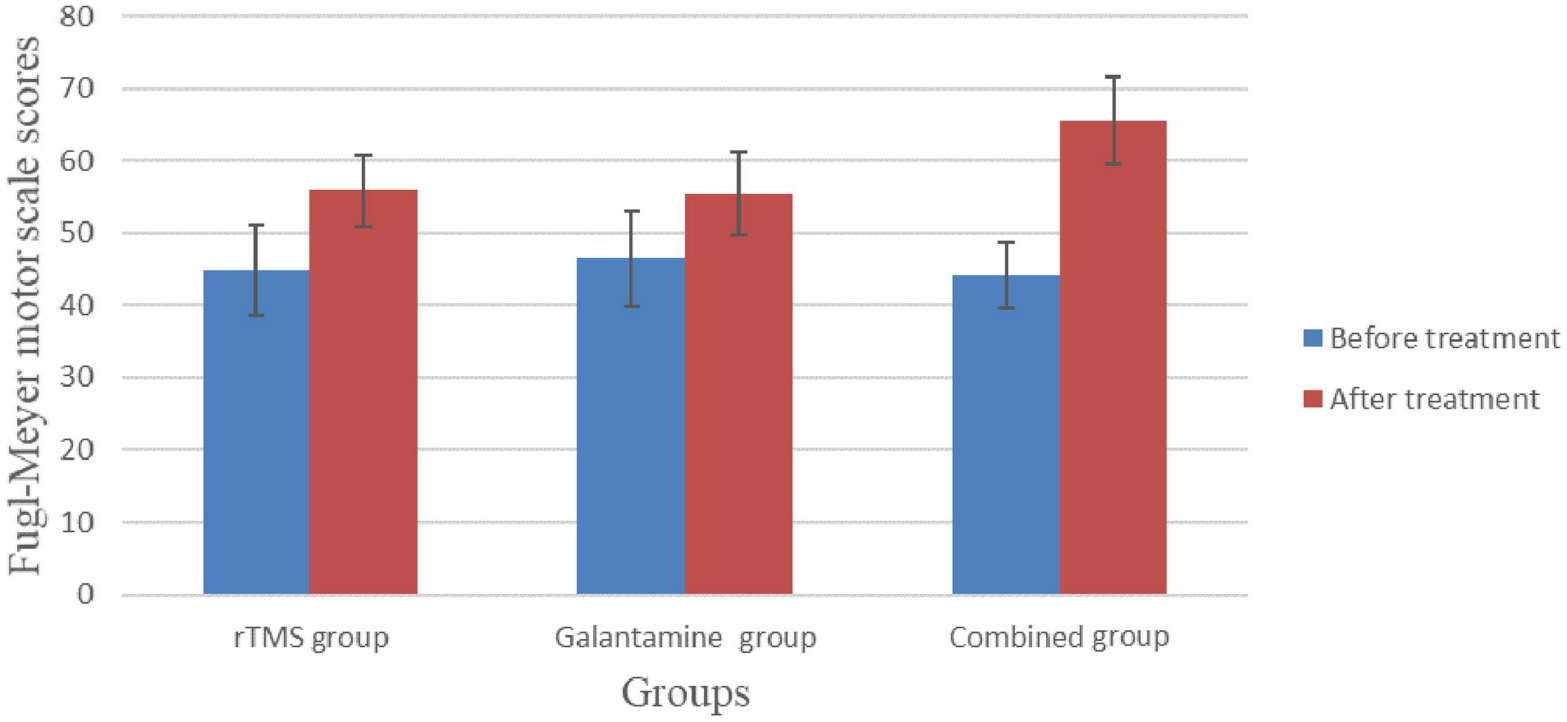
Comparison of Fugl-Meyer motor scale scores before and after treatment in the three groups.

Before treatment, there was no significant difference in Fugl-Meyer scores among the three groups (*p* > 0.05). After four weeks of treatment, the scores of all groups were improved, and the difference was statistically significant (all *p* < 0.05). There were significant differences between the groups, with the highest score in the combined treatment group (*p* < 0.05) (Table 3; Figure 4).

**Table 3:** Comparison of Fugl-Meyer motor scale scores before and after treatment in the three groups (points, $\bar{x}\pm s$ ).

Group	Number	Before treatment	After treatment	*T* value	*p* value
rTMS group	30	44.87 ± 6.22	55.87 ± 4.88	−8.30	0.00
Galantamine group	30	46.53 ± 6.63	55.43 ± 5.72	−6.42	0.00
Combined group	30	44.17 ± 4.53	65.50 ± 6.05	18.10	0.00
*χ^2^/F* value		1.29	31.28		
*p* value		0.28	0.00		

### 3.4 The modified Barthel index before and after treatment in the three groups of patients to assess the ability of daily living

**Figure 5:**
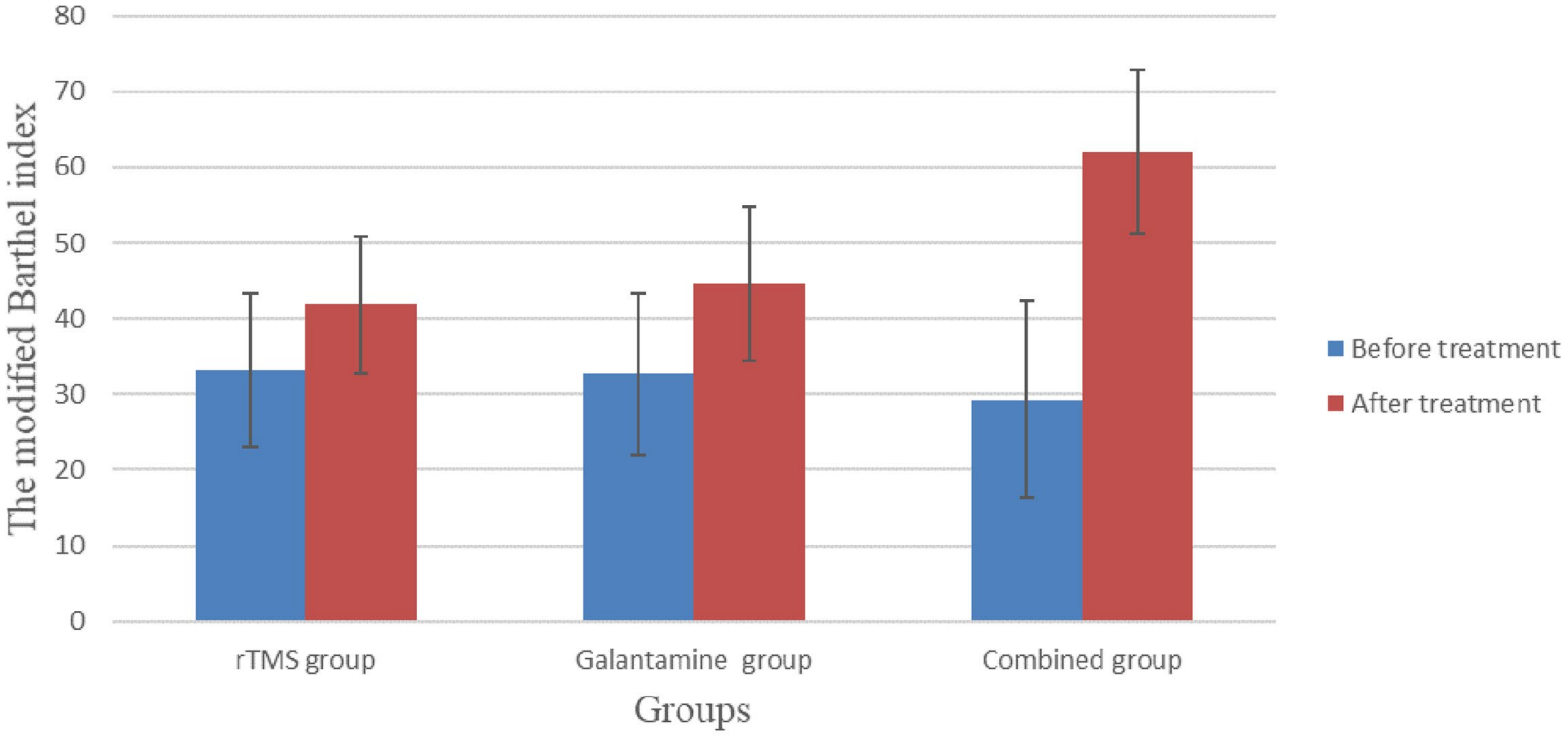
The modified Barthel index before and after treatment in the three groups to assess the ability of daily living.

Before treatment, there was no significant difference in modified Barthel index scores among the three groups (*p* > 0.05). After four weeks of treatment, the scores of all groups were improved, and the difference was statistically significant (all *p* < 0.05). There were significant differences between the groups, with the highest score in the combined treatment group (*p* < 0.05) (Table 4; Figure 5).

**Table 4:** The modified Barthel index before and after treatment in the three groups to assess the ability of daily living (points, $\bar{x}\pm s$ ).

Group	Number	Before treatment	After treatment	*T* value	*p* value
rTMS group	30	33.17 ± 10.21	41.83 ± 9.00	−3.50	0.02
Galantamine group	30	32.67 ± 10.73	44.67 ± 10.17	−4.94	0.00
Combined group	30	29.33 ± 13.05	62.00 ± 10.80	−11.03	0.00
*χ^2^/F* value		1.00	35.76		
*p* value		0.37	0.00		

### 3.5 Comparison of serum Hcy and NSE levels in the three groups of patients before and after treatment

**Figure 6:**
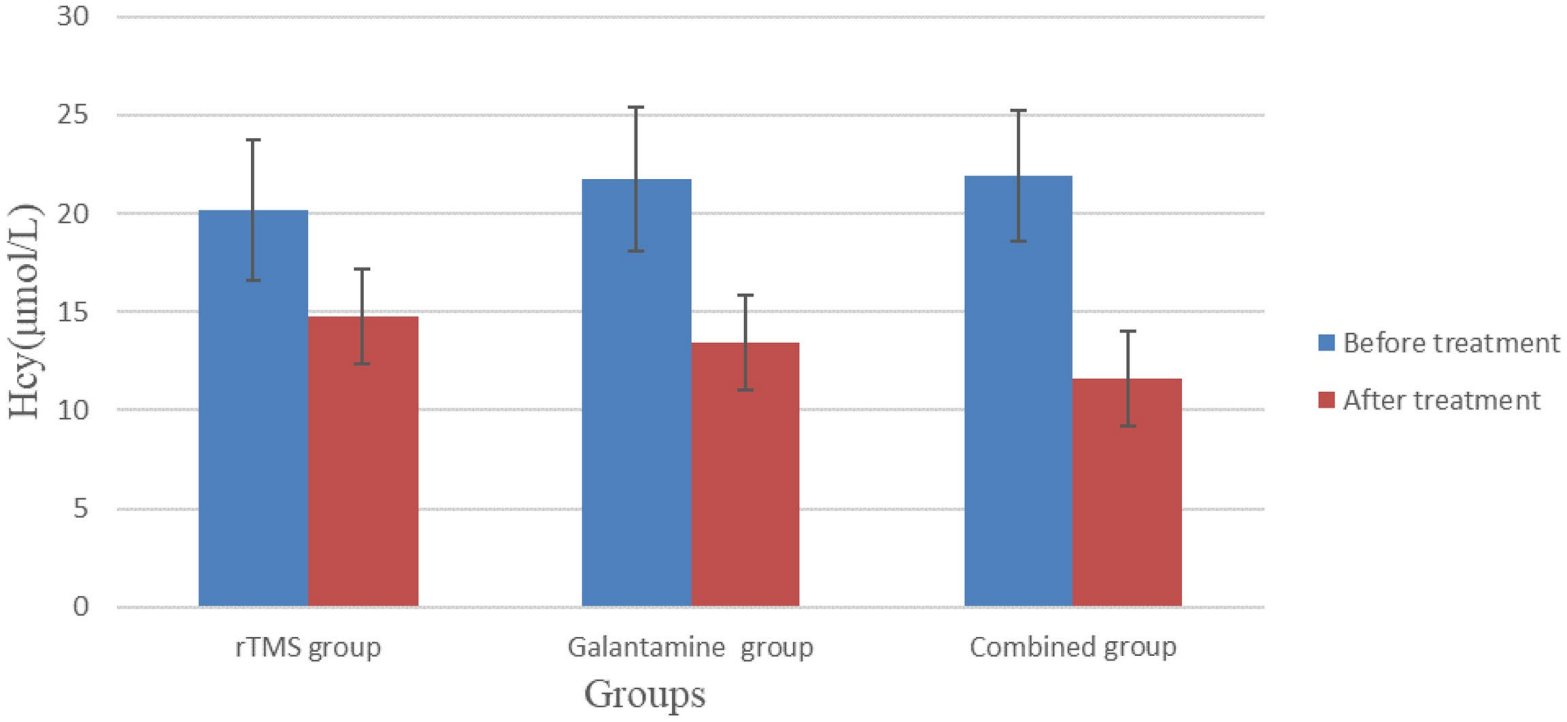
Comparison of serum Hcy levels in three groups of patients before and after treatment.

**Figure 7:**
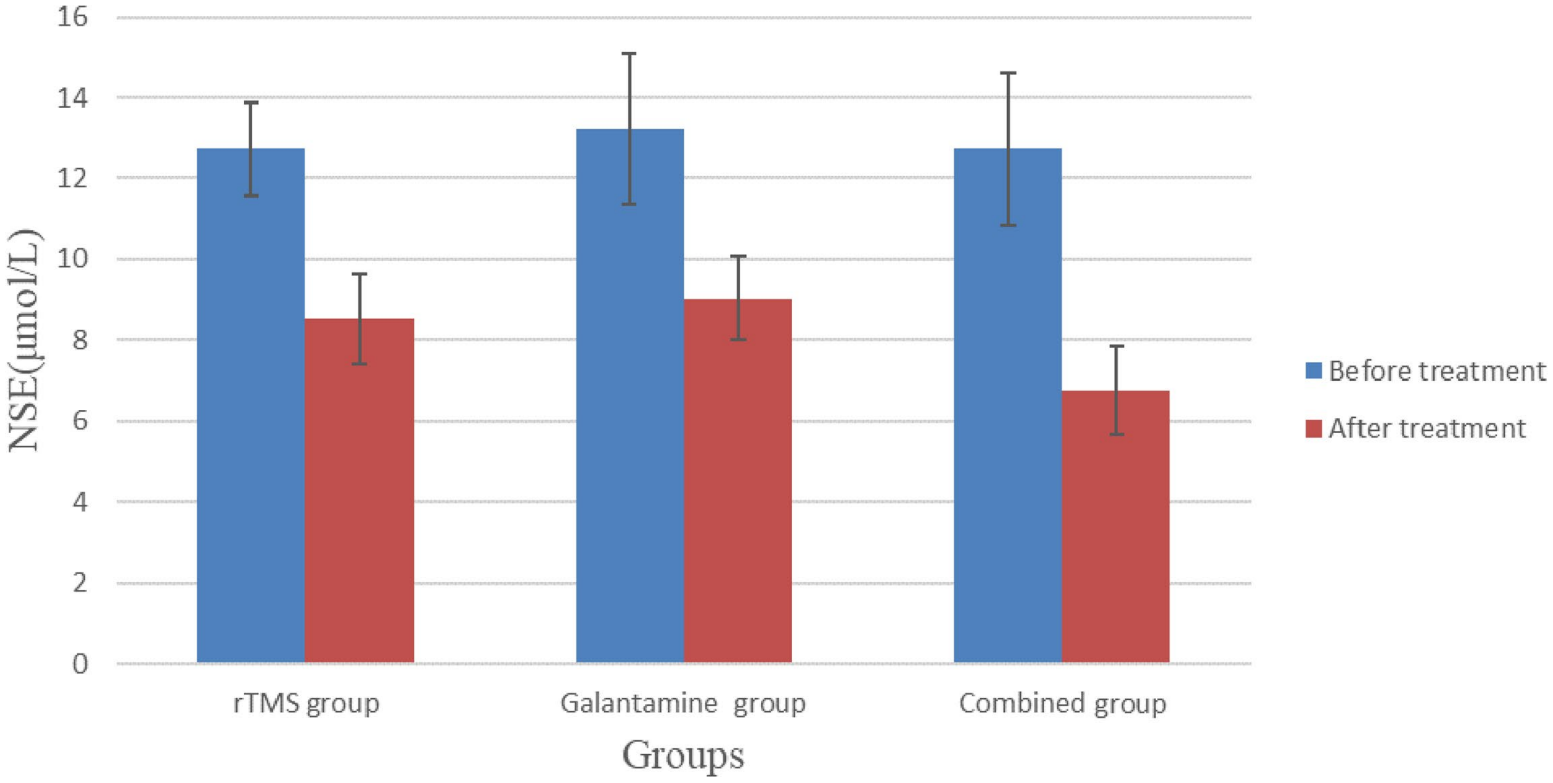
Comparison of serum NSE levels in three groups of patients before and after treatment.

Before treatment, serum Hcy and NSE levels in the three groups were not statistically significant (*p* > 0.05). However, after 4 weeks of treatment, serum Hcy and NSE levels in the three groups were lower than before treatment, and the differences were statistically significant (*p* < 0.05), with the combined group having the lowest level (Table 5; Figures 6, 7).

**Table 5:** Comparison of serum Hcy and NSE levels in three groups of patients before and after treatment.

Group	Number	Hcy (μmol/L)		NSE (μmol/L)	
		Before treatment	After treatment	Before treatment	After treatment
rTMS group	30	20.20 ± 3.57	14.75 ± 2.42^a^	12.73 ± 1.15	8.52 ± 1.13^a^
Galantamine group	30	21.74 ± 3.66	13.46 ± 2.43^a^	13.23 ± 1.85	9.04 ± 1.04^a^
Combined group	30	21.89 ± 3.33	11.62 ± 2.37^a^	12.73 ± 1.87	6.77 ± 1.10^a^
*χ^2^/F* value		2.13	12.83	0.80	35.56
*p* value		0.13	0.00	0.46	0.00

a Compared with before treatment, *p* < 0.05.

## 4 Discussion

Our research results showed that after 4 weeks of treatment, the ADAS-cog, MMSE, MoCA, Fugl-Meyer scale scores, and modified Barthel index scores of patients in the combined experimental group were improved to the greatest extent, while NSE and HCY were also significantly decreased. The improvement of cognitive function promoted the improvement of motor function and the enhancement of the self-care ability of daily life to a greater extent.

Homocysteine (Hcy) is an intermediate product of methionine metabolism. Under physiological conditions, its plasma concentration is maintained at a low level, and an increase in its level can increase the risk of diabetes, coronary heart disease, and cerebrovascular disease. Also, it is an independent risk factor for dementia (37, 38). Many studies have shown that Hcy levels in patients with post-stroke cognitive impairment are significantly higher than those in stroke patients without cognitive impairment, supporting Hcy as a biomarker and predictor of PSCI (39). Studies have shown that patients with higher Hcy levels have greater cerebral cortical and hippocampal atrophy than patients with lower Hcy levels (40). Given the important role of the hippocampus in cognitive memory, this could lead to more severe cognitive impairment.

Neuron-specific enolase (NSE) is specifically distributed in nerve tissue and neuroendocrine cells. When nerve cells are injured by ischemia, the permeability of the nerve cell membrane changes and NSE can be released from the cells into the peripheral blood circulation. It can be used to assess the extent of damage to the biofilm structure of the central nervous system as a marker of neuronal damage. Studies have shown that serum NSE levels are positively correlated with the size of cerebral infarction and promote local neuroinflammatory response, which is an indicator to judge the severity of brain injury (41). Recent studies have shown that NSE is closely related to stroke prognosis and is a reliable indicator of stroke functional prognosis (42). The level of serum NSE is a sensitive biochemical index for judging the degree of brain nerve damage in patients with vascular dementia. The severity and prognosis of dementia can have an effective predictive role (43).

Our experiment showed that the combination of high-frequency TMS and galantamine had the greatest reduction in HCY and NSE, which minght indicate that neuronal damage had been repaired and greatly protected.

According to research reports, diet and poor lifestyle habits can affect the emotional and cognitive function of people with vascular dementia. The study by Fisicaro et al. (44) suggested that among Italian elderly people with subcortical ischemic vascular disease, daily mocha coffee intake was associated with higher cognitive and emotional states, and there was a significant dose–response relationship even with moderate consumption. And among non-smokers with mild vascular cognitive impairment, high coffee intake was associated with better mental cognitive status (45).

When TMS is used to detect brain physiological changes in VCI patients, it cannot be ignored that there are gender differences in these brain physiological changes. Cantone et al. (46) showed that in VCI patients, men scored worse on the overall cognitive test, executive function and independence, and the latency of motor evoked potential (MEP) in both sides of men was significantly longer, as was the central motor conduction time (CMCT) and central motor conduction time of F wave (CMCT-F) in the left hemisphere, while the short-interval intracortical inhibition (SICI) in the right hemisphere was lower. However, in our study, no gender differences were found in the indicators detected by transcranial magnetic resonance imaging. This may be related to race, testing techniques, accompanying disease effects, and sample size selection.

The mechanism of repetitive transcranial magnetic stimulation to improve cognitive impairment is complex and has multiple effects. One of the possible mechanisms is that rTMS activates a rich cholinergic neural network in the brain after stimulating DLPFC. About 20-25 ms before giving motor cortex stimulation, give an electrical stimulation to the wrist of the median nerve, then the amplitude of the induced motor evoked potential will generally be suppressed. This phenomenon is called short latency afferent inhibition (SAI). This parameter is considered to reflect central cholinergic activity. In the recognition memory stage of healthy people, SAI is significantly enhanced compared with resting time, and it can be regulated by the ongoing memory process, showing that normal memory processes are closely related to cholinergic neuron activity (47). In AD patients, multiple studies have reported a decrease in SAI, indicating that the central cortical–cortical cholinergic circuit may be damaged (48, 49). After transcranial magnetic stimulation treatment, these patients showed an increase in SAI accompanied by improvement in cognitive assessment. Short latency afferent inhibition (SAI) is thought to be mediated by cholinergic projections on M1 and is considered a putative marker of central cholinergic activity. Homotaurine is a Gamma-Amino Butyric Acid (GABA) partial agonist. There are reports that homotaurine can improve cholinergic transmission by modulating inhibitory cortical activity, but it has no effect on the neuropsychological scores of patients after 4 weeks of short-term treatment (50). Whether drugs that directly regulate SAI can improve cognitive function in the long term remains to be explored.

Another possible mechanism by which transcranial magnetism improves cognitive function is the mechanism of long-term enhanced plasticity. Transcranial magnetism in healthy musicians and athletes can improve learning and exercise performance by promoting LTP-like activation of key networks (51). Li et al.’s (52) study showed that using transcranial magnetic stimulation at a frequency of 20 Hz to the dorsolateral prefrontal cortex (DLPFC) of Alzheimer’s patients significantly increased the amplitude of motor evoked potentials at different time periods, with a significant difference in change rate compared to the control group. Moreover, the improvement of cortical plasticity was significantly positively correlated with cognitive changes. Similar therapeutic responses have also been observed in animal experiments, showing that the effect of rTMS stimulation on recognition and memory ability in mice is positively correlated with the induction of hippocampal LTP *in vivo*, and its therapeutic effect is closely related to the intensity of stimulation (53).

Cortical LTP-like plasticity can not only be used as an indicator of therapeutic efficacy, but also as a parameter to predict cognitive status. Di Lorenzo et al.’s study showed that patients with mild cognitive impairment had moderate but significant LTP-like cortical plasticity impairment, and this impairment was more severe in AD patients with dementia. LTP-like cortical plasticity can be used as a new biomarker to predict clinical progression in patients with memory impairment in the prodromal stage of AD (54). The research results of Buss et al. showed that compared with the control group, MCI patients lacked ltp like neural regulation, which was not related to the quantitative amyloid protein load displayed by positron emission tomography. Surprisingly, a larger ltp like response was associated with poorer memory function in the MCI group, highlighting the complex role of neural plasticity in the prodromal stage of AD (55). Cortical plasticity is influenced by multiple factors. Koch et al.’s (56) study showed that cerebrospinal fluid Tau levels affect cortical plasticity in Alzheimer’s disease patients. Higher CSF t-Tau levels are associated with stronger inhibition of motor evoked potentials, but are associated with cerebrospinal fluid amyloid-β (A) (β) Level independent.

Furthermore, the mechanism of repeated transcranial magnetic therapy may also include inhibiting local neuroinflammatory responses in brain injury. Chen et al. (57) showed that 20 Hz high-frequency rTMS could attenuate white matter lesions in rats with ischemic stroke, reduce the levels of pro-inflammatory cytokines, and increase anti-inflammatory cytokines. It also decreased the number of CD68- and CD16/32-positive microglia and increased the number of CD206-positive microglia, while expressions of p-JAK2, JAK2, p-STAT3, and STAT3 were increased. The underlying mechanism may be related to the regulation of the JAK2-STAT3 pathway. In addition, the neurological deficit score of the rats was reduced, and the cognitive function of rats with lower-frequency stimulation was further improved. Hong et al. (58) conducted rTMS treatment on rats with transient middle cerebral artery occlusion (tMCAO) model to understand the effect of rTMS on PSCI. The results indicated that high-frequency rTMS could significantly improve neurological and cognitive function, according to mNSS and MWM tests. In addition, they found 85 differentially expressed genes, including 71 upregulated genes and 14 downregulated genes, between the rTMS group and tMCAO group. These results demonstrate that rTMS has a beneficial effect on PSCI, and its mechanism may be related to the regulation of synaptic plasticity and functional genes such as Calb2, Zic1, Kcnk9, and Grin3a in the hippocampus.

The mechanism of action of rTMS also includes promoting white matter growth and repair, improving brain metabolism, anti-apoptosis, improving ischemic tolerance of nerve block, and promoting cerebral blood supply (59). In all, preliminary experimental studies indicate a complex scenario potentially relevant to the therapeutic effects of NIBS, including gene activation/regulation, *de novo* protein expression, morphological changes, changes in intrinsic firing properties and modified network properties resulting from changed inhibition, homeostatic processes and glial function (60). rTMS has the advantages of non-invasiveness, no pain, strong penetrating power, easy operation, safety, reliability, etc. It has also been applied in many fields of basic neuroscience and clinical practice.

The research results show that galantamine combined with rTMS can effectively improve the cognitive function of stroke patients and promote the repair of nerve damage. The improvement of cognitive function can synergistically promote the improvement of patients’ limb functions, improve the self-care ability of daily life, and enable patients to achieve optimal benefit. Studies have shown that rTMS can increase blood flow in the brain’s frontal cortex, reduce damage to cholinergic neurons, increase the content of energy-supplying substances such as adenosine triphosphate, and improve the utilization of glucose in damaged brain tissue (38).

The pharmacological mechanism of Galantamine mainly includes inhibition of cholinesterase, regulation of nicotinic cholinergic receptor structure (allosteric regulation), protection of hippocampal neurons, and improvement of cerebral blood supply. After rTMS treatment, the central rich cholinergic nervous system can be widely activated, and we speculate that galantamine may help maintain this activation state more persistently, jointly promoting the improvement of cognitive function in PSCI patients. The combination of the two produces a synergistic effect, which can maximize the cognitive function of patients, promote the improvement of limb function, improve the self-care ability of daily life, and reduce the burden on families and society.

Current research shows that galantamine can improve the cognitive function and activities of daily living of vascular cognitive impairment and promote the comprehensive recovery of patients’ functions. In clinical application, the drug dose is small, the adverse reactions are minor, and the drug is considered to be safe and financially convenient. Due to the inhibition of cholinesterase by Galantamine, the adverse reactions mainly include gastrointestinal reactions, the most common ones being nausea, vomiting, diarrhea, and constipation. Nonetheless, most of these effects are mild and transient. Our research also showed that in the control group, after oral administration of Galantamine Hydrobromide Tablets and conventional rehabilitation treatment, the evaluation of the cognitive function of patients after treatment was significantly higher than before treatment, the curative effect was good, and no significant adverse reactions were observed.

At present, there are still some unclear issues in rTMS treatment. Different types of dementia (such as Alzheimer’s disease, vascular dementia, Louis body dementia, etc.) have different pathological characteristics and pathogenesis. Dementia is a progressive disease with varying pathological features and cognitive impairment levels at different stages. Different patients have different genetic backgrounds, dietary habits, comorbidities, and other factors. These factors may all affect the therapeutic effect of TMS. The treatment plan for TMS requires in-depth research on different types and stages of dementia and individual differences, and the development of personalized and specific treatment plans in order to achieve the best results.

There are differences in the therapeutic effects of different TMS stimulation schemes on different diseases. For example, the review by Lanza et al. (61) shows that different frequencies of stimulation are applied to different areas (such as the motor cortex, DLPFC, anterior parietal lobe, etc.), resulting in different therapeutic effects in different diseases. Different stimulation schemes should be used for neurodegenerative diseases, neuroinflammation, nerve injury, developmental disorders, and other diseases.

At the same time, TMS detection indicators such as SAI vary greatly in patients with vascular dementia, which may affect the reliability of their indicators.

At present, a single detection method cannot accurately predict and evaluate the development of dementia. The comprehensive use of various modern neurophysiological detection techniques, such as high-density electroencephalography, event-related potentials, TMS, biomarkers, and structural and functional neuroimaging, may enable the identification of high-risk populations with high specificity and early intervention measures to block the development of cognitive impairment (62). This comprehensive method can also provide reliable tools for drug research, brain connectivity network detection, and neural regulation technology research.

The DLPFC is a core region involved in executive functions such as working memory and cognitive flexibility. Stimulation of DLPFC with rTMS significantly improves memory function in patients with VD or AD. This may be related to the fact that DLPFC promotes the formation of long-term memory by interacting with regions within the medial temporal lobe network (such as the hippocampus) during memory encoding and retrieval (63). Most studies on rTMS and brain cognitive function focus on left, right or bilateral DLPFC and right inferior frontal gyrus (IFG) (17, 18, 63). However, cognitive function is a high-level function of the brain, which involves the complex comprehensive and coordinated execution of functions among multiple brain regions and neural networks. The role of other rTMS stimulation regions (such as cerebellum, precuneus and parietal regions) in improving brain cognitive function needs further research in the future (64).

rTMS, as one of the most important non-invasive treatment methods, has precise efficacy and few side effects, and is an important tool for clinical treatment and basic research.。In future, with the development of transcranial magnetic technology, people may also use it as an instrument for screening of population at risk, studying specific drug-induced changes in the electrical properties of the human cortex, evaluating drug efficacy, detecting brain connection models, and testing neuromodulation therapeutic tools to promote cognitive recovery.

The present study has some limitations. Our research objectives were limited to patients within 6 months after stroke. The effects of Galantamine combined with transcranial magnetism on patients with a longer course of the disease need to be further investigated, and the most appropriate course of treatment also needs to be explored. Due to limited research funding, this study was unable to observe long-term effects, and efforts will be made to pay more attention in the future. The cellular and molecular mechanism of combination therapy also needs to be further developed in related basic research and animal research.

## Data availability statement

The original contributions presented in the study are included in the article/supplementary material, further inquiries can be directed to the corresponding authors.

## Ethics statement

The studies involving humans were approved by Shanghai Second Rehabilitation Hospital. The studies were conducted in accordance with the local legislation and institutional requirements. The participants provided their written informed consent to participate in this study.

## Author contributions

GH: Conceptualization, Methodology, Project administration, Writing – original draft, Writing – review & editing. LZ: Data curation, Formal analysis, Writing – original draft, Writing – review & editing. XS: Conceptualization, Data curation, Formal analysis, Writing – original draft, Writing – review & editing. LW: Conceptualization, Data curation, Formal analysis, Writing – original draft, Writing – review & editing. QX: Formal analysis, Methodology, Writing – original draft, Writing – review & editing. QL: Data curation, Project administration, Writing – original draft, Writing – review & editing. WH: Data curation, Investigation, Writing – original draft, Writing – review & editing. YX: Data curation, Formal analysis, Writing – original draft, Writing – review & editing.
